# The Effects of INDY on Fly Metabolism

**DOI:** 10.1093/geroni/igaa057.2622

**Published:** 2020-12-16

**Authors:** Blanka Rogina, Kavitha Kannan, Dushyant Mishra, Jacob Macro, Danielle Lesperance, Nichole Broderick, Shivani Padhi, Aaron Rosenbloom-Snow

**Affiliations:** 1 UConn Health, Farmington, Connecticut, United States; 2 University of Connecticut, Storrs, Connecticut, United States

## Abstract

The Indy (I’m not dead yet) gene encodes a plasma membrane citrate transporter in Drosophila. INDY reduction affects metabolism and extends longevity of flies and worms. In flies, INDY is predominantly expressed in the midgut, fat body and oenocytes, tissues with a key role in metabolism. We hypothesize that INDY reduction in the midgut regulates citrate levels leading to metabolic changes that preserve intestinal stem cell (ISC) homeostasis and slows aging by modifying Insulin/Insulin-like signaling (IIS), which is a key nutrient sensing pathway. Our second goal was to examine the role of JAK/STAT signaling pathway, which activates epithelial renewal in the gut, in response to aging-related stressors. We hypothesize that Indy reduction has effects on the microbiome, preventing bacterial overgrowth and altering community diversity, leading to extended longevity in a JAK/STAT-mediated fashion. Our data suggest that effects of Indy reduction is mediated by reduced IIS and JAK/STAT pathways

